# Diagnostic Value of Metagenomic Next-Generation Sequencing for the Detection of Pathogens in Bronchoalveolar Lavage Fluid in Ventilator-Associated Pneumonia Patients

**DOI:** 10.3389/fmicb.2020.599756

**Published:** 2020-12-01

**Authors:** Xiaowei Fang, Qing Mei, Xiaoqin Fan, Chunyan Zhu, Tianjun Yang, Lei Zhang, Shike Geng, Aijun Pan

**Affiliations:** ^1^ Department of Intensive Care Unit, The First Affiliated Hospital of USTC, Division of Life Science and Medicine, University of Science and Technology of China, Hefei, China; ^2^ Department of Intensive Care Unit, The Affiliated Provincial Hospital of Anhui Medical University, Hefei, China

**Keywords:** metagenomics next-generation sequencing, bronchoalveolar lavage fluid, ventilator-associated pneumonia, mixed infection, diagnostic value, intensive care unit

## Abstract

**Objective**: To evaluate the diagnostic performance of metagenomic next-generation sequencing (mNGS) using bronchoalveolar lavage fluid (BALF) in patients with ventilator-associated pneumonia (VAP).

**Methods**: BALF samples of 72 patients with VAP were collected from August 2018 to May 2020. The diagnostic performance of conventional testing (CT) and mNGS methods were compared based on bacterial and fungal examinations. The diagnostic value of mNGS for viral and mixed infections was also analyzed.

**Results**: The percentage of mNGS positive samples was significantly higher than that estimated by the CT method [odds ratio (OR), 4.33; 95% confidence interval (CI), 1.78–10.53; *p* < 0.001]. The sensitivity and specificity of mNGS for bacterial detection were 97.1% (95% CI, 93.2–101.0%) and 42.1% (95 CI, 30.7–53.5%), respectively, whereas the positive predictive value (PPV) and the negative predictive value (NPV) were 60.0% (95% CI, 48.7–71.3%) and 94.1% (95% CI, 88.7–99.6%), respectively. A total of 38 samples were negative for bacterial detection as determined by the CT method, while 22 samples were positive as shown by the mNGS method. Conflicting results were obtained for three samples between the two methods of bacterial detection. However, no significant differences were noted between the mNGS and CT methods (OR, 1.42; 95% CI, 0.68–2.97; *p* = 0.46) with regard to fungal infections. The sensitivity and specificity of mNGS were 71.9% (95% CI, 61.5–82.3%) and 77.5% (95% CI, 67.9–87.1%), respectively. mNGS exhibited a PPV of 71.9% (95% CI, 61.5–82.3%) and an NPV of 77.5% (95% CI, 67.9–87.1%). A total of 9 out of 40 samples were found positive for fungi according to mNGS, whereas the CT method failed to present positive results in these samples. The mNGS and CT methods produced conflicting results with regard to fungal detection of the two samples. A total of 30 patients were virus-positive using mNGS. Furthermore, 42 patients (58.3%) were identified as pulmonary mixed infection cases.

**Conclusions**: mNGS detection using BALF improved the sensitivity and specificity of bacterial identification in patients who developed VAP. In addition, mNGS exhibited apparent advantages in detecting viruses and identifying mixed infections.

## Introduction

Ventilator-associated pneumonia (VAP) induced following mechanical ventilation remains a major health condition, which has to be treated by intensive care unit (ICU) physicians. VAP exhibits high morbidity and mortality, despite the advances made in antibiotic therapy and the multitude of strategies used for VAP prevention. It has been reported that the incidence of VAP in ICU is 9–27%, whereas the mortality rate ranges between 30 and 70% (Chastre and Fagon, 2002; Valles et al., 2007; Rosenthal et al., 2020). A high number of pathogens have been associated with VAP. In the majority of the cases, VAP patients undergo endotracheal intubation and bronchoalveolar lavage fluid (BALF) becomes a more accessible clinical specimen with a higher diagnostic value than other specimens. Conventional testing (CT) methods of BALF, including bacterial and fungal smear and culture have been routinely performed to assess the microbiological information. However, these methods of assessment are time-consuming and exhibit a low positive rate, which cannot meet the diagnostic needs of a critically ill patient. Culture-independent CT methods (serological testing and nucleic acid amplification testing) have proven useful for detecting viruses. However, due to the limited detection range of the PCR kits, the combination of several diagnostic methods that can complement the viral detection is essential.

Metagenomic next-generation sequencing (mNGS) provides an unbiased approach, which allows universal pathogen detection from clinical specimens and can therefore be considered as an ideal method for detecting bacteria, fungi, and viruses (Goldberg et al., 2015; Miao et al., 2018). It has been suggested that the mNGS can be used for the diagnosis of severe pneumonia patients and for providing guidance of their clinical treatment, which may lead to the reduction of the mortality rate (Xie et al., 2019). Moreover, increasing numbers of rare pathogens have been detected by the mNGS method, which provides a rapid and effective strategy for the diagnosis of certain difficult cases (Chiu et al., 2017; Ai et al., 2018; Zhang et al., 2019; Wang et al., 2020). However, the application of mNGS on VAP patients in general ICU has been examined to a lesser extent. Moreover, results showed a large difference in pathogens among different clinical departments due to different sources. Furthermore, the majority of the mNGS studies have focused on the diagnostic advantage of specific pathogens. The mNGS method has been shown to be superior to the culture methods used for identification of *Streptococcus pneumoniae*, *Haemophilus influenza*, and *Prevotella melaninogenicus*; however, no significant difference was reported between the two techniques with regard to fungal detection (Xie et al., 2019). Conversely, contradictory opinions have also been reported (Toma et al., 2014; Miao et al., 2018). In the present study, the mNGS data of 72 BALF samples were summarized and the diagnostic performance of the mNGS method was compared to that of the CT methods with regard to bacterial and fungal detection, respectively. Moreover, the diagnostic value of mNGS for viral and mixed infections was analyzed.

## Materials and Methods

### Study Patients

**Figure 1:**
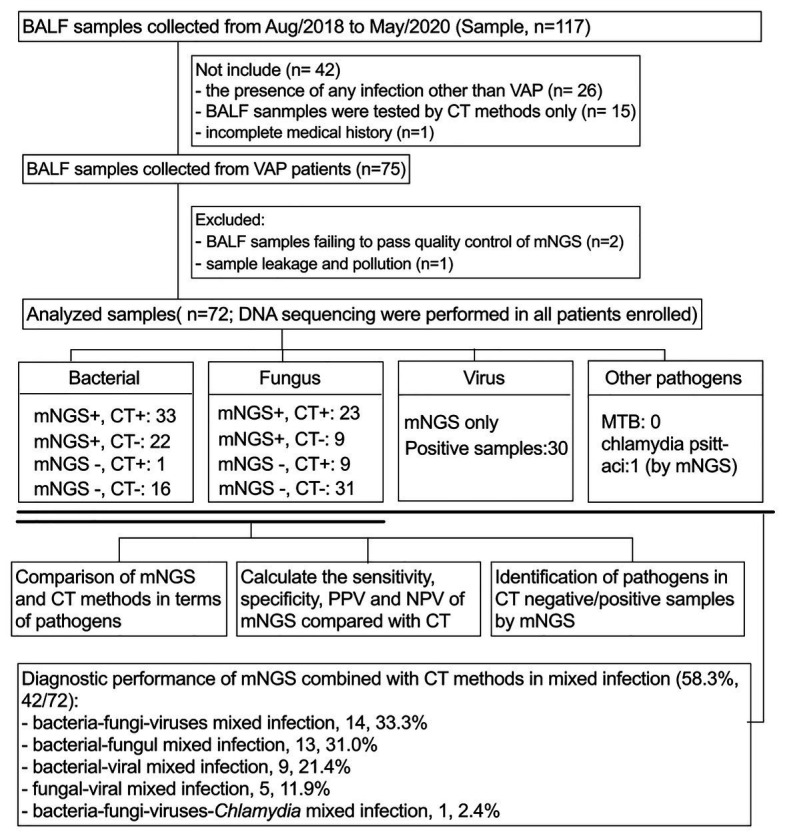
Flow chart of sample screening. Seventy-two samples were included in this study. All pathogens are classified as bacteria, fungi, viruses, and others. The sensitivity, specificity, PPV, and NPV of bacterial and fungal pathogens using mNGS and CT methods were compared in a paired format and, respectively, analyze the diagnostic performance of mNGS in CT-negative/positive samples. In addition, the identification performance of mNGS on mixed infections was also analyzed. BALF, bronchoalveolar lavage fluid; mNGS, metagenomics next generation sequencing; CT, conventional testing; PPV, positive predictive value; NPV, negative predictive value.

A retrospective analysis of VAP patients was performed at 130-bed ICU in The First Affiliated Hospital of USTC (Anhui Provincial Hospital) from August 2018 to May 2020 (Figure 1). The following inclusion criteria were used: (i) mechanical ventilation (>48 h) and diagnosis with VAP according to the diagnostic criteria recommended by the VAP expert meeting of American Thoracic Society (Society and America, 2005); (ii) all patients underwent bronchoscopy to obtain BALF; (iii) both mNGS and CT methods were used to detect pathogens; (iv) CT methods included at least bacterial and fungal smear and culture, Grocott’s methenamine staining and acid-fast staining; and (v) routine blood and inflammatory markers, which included CRP, PCT, (1, 3)-β-d-glucans, and galactomannan antigen were tested within 24 h prior to or following BALF collection. TB-spot and GeneXpert were also included for the detection of tuberculosis (TB). The following exclusion criteria were used: (i) the presence of any infection other than VAP acquired during ICU stay; (ii) BALF samples that failed to pass quality control of mNGS (e.g., the human origin sequences over 99%); (iii) incomplete clinical history; and (iv) sample leakage and contamination. Baseline data were collected from the electronic medical records of the patients, including demographic characteristics, comorbidities, immunosuppressive state, treatment process, and prognosis.

### Sample Acquisition, Processing, and Nucleic Acid Extraction

BALF samples were collected within 24–48 h post-VAP diagnosis. The bronchoscope was wedged in a bronchus from the lesion site under sterile operating conditions and 100 ml of sterile saline solution was instilled at 37°C in the associated bronchial tube. Approximately 5 ml BALF sample was retrieved and placed into sterile containers. If scattered lesions were present, BALF was collected from the right middle lobe or the sub-segment of the left lingual lobe. The BALF samples were divided into equal aliquots. The CT methods were performed by culture of the bacteria in blood agar, MacConkey agar, and chocolate agar plate at 35°C for a maximum period of 5 days, whereas the fungi were cultured in Sabouraud agar at 35°C for a maximum period of 5 days. The organisms were identified using an automated system (Vitek 2 automated system, bioMérieux, Marcy-l’ Etoile, France). The bacteria were identified using colony morphology and Gram staining, whereas the filamentous fungi were identified by smear results. A different aliquot was used for mNGS. Initially, a 1.5-ml microcentrifuge tube (containing 0.5 ml BALF sample and 1 g 0.5-mm glass beads) was connected to a horizontal platform on the vortex mixer. Subsequently, the tube was agitated vigorously at 2,800–3,200 rpm for approximately 30 min. Subsequently, the 0.3 ml sample was separated into a new 1.5 ml microcentrifuge tube and DNA was extracted by the TIANamp Micro DNA Kit (DP316, Tiangen Biotech).

### Library Construction and Sequencing

The extracted DNA was sonicated and 200-500 BP DNA fragments were obtained. These DNA fragments were broken by ultrasound or fragmentary enzyme and were subjected to terminal repair, phosphorylation, and A-tailing reaction. The BGISEQ-500 platform-specific adaptors were ligated to the A-tailed fragments and the ligated fragments were purified and subsequently amplified by PCR. DNA nanoballs (DNB) were prepared using single-stranded DNA circles. Finally, each DNB was loaded into 1 lane for sequencing. The sequencing was performed on the BGISEQ-500 platform (Fang et al., 2017).

### Bioinformatic Analyses

Initially, the removal of low quality and short sequences (<35 bp) was performed to obtain high-quality sequencing data. Subsequently, human sequence data were identified and excluded by mapping on the human reference (hg19) using burrows Wheeler aligner software. Following completion of the above steps, the remaining sequence data were aligned to the current bacterial, viral, fungal, and protozoan databases, which were downloaded from the National Biotechnology Center.^1^ The database used for the present study contained 2,328 bacterial species, 4,189 viral species, 199 fungal species, 135 parasites, and 40 mycoplasma/Chlamydia, which were associated with human diseases.

### Identification of Pathogens

#### Pathogens Identified by CT Methods

(i) **Bacteria**: A bacterial number higher than 10^4^ CFU/ml was considered a positive criterion for culture. Positive BALF smear results were defined as a Gram-positive and/or Gram-negative bacterium detected by microscopic investigation; (ii) **Fungi**: the “definitive diagnosis” required culture and smear of fungi from a BALF sample, while the following factors required consideration in confirming the “clinical diagnosis”: host factors, clinical characteristics, and microbiological evidence. In addition, the (1, 3)-β-D-glucan and galactomannan antigen detection may be of important auxiliary diagnostic value in fungal detection; pneumocystis was confirmed by Grocott methenamine staining of BALF samples. (iii) **Virus**: the target virus was defined by PCR detection in BALF samples; and (iv) **Tuberculosis**: the definitive diagnosis of pulmonary TB was based on sputum smear examinations for acid-fast bacilli and/or the culture of TB. The diagnosis of pulmonary TB was established by TB-associated symptoms, along with a computed tomography scan and the results of the TB-spot and/or GeneXpert.

#### Criteria for a Positive mNGS Result

(i) the relative abundance of pathogens detected by mNGS was higher than 30% at the genus level irrespective to the results of the CT method; (ii) CT and mNGS identified the same microorganisms and the number of unique reads was more than 50 from a single species; (iii) if only the microorganisms were identified by mNGS, they were considered as new potential pathogens; and (iv) TB was considered positive when at least 1 read was mapped to either the species or the genus level. (Li et al., 2018)

Infectious pathogens were defined if any of the above conditions were met. Furthermore, the mixed infection was defined as the isolation of more than one pathogenic species. It needs to be emphasized that microorganisms could not be directly judged as infection, colonization, and pollution by CT and mNGS results. Therefore, after obtaining CT and mNGS results, clinical features were taken into consideration by two physicians to identify the pathogens and reached a consensus.

### Statistical Analysis

Continuous variables with normal distribution were expressed as mean ± standard deviation (SD), whereas continuous data with non-normal distribution were expressed as median [first quartile(Q1), third quartile(Q3)] and categorical data were expressed as numbers (percentage). We used conventional testing results as reference standard to evaluate the diagnostic efficacy of mNGS. According to the extracted data, a 2 × 2 contingency table was established to calculate sensitivity, specificity, positive predictive value (PPV), and negative predictive value (NPV). The SPSS 22.0 software was used for data analysis and a two-tailed value of *p* of 0.05 was considered for significant differences.

## Results

### Demographic Characteristics

The clinical features of the 72 patients with VAP, including demographic characteristics, disease severity, comorbidities, treatment, and prognosis are presented in Table 1. In the present study, all enrolled patients were subjected to only one-time detection by the mNGS method. A total of 29 out of 72 patients were female, whereas 43 were male, with a median age of 62.0 years. The median PSI scores were 110.0. In addition, the proportion of patients with immunosuppression was 37.5% (27/72). All patients with evidence of VAP, based on Computed Tomography scans, who received antibiotic therapy prior to mNGS were enrolled in the present study. The median duration of mechanical ventilation prior to the mNGS procedure was 8.0 days. The overall ICU mortality was 52.8%.

**Table 1:** Clinical characteristics of 72 patients included.

Characteristics	72 patients
Age, years, median (Q1, Q3)	62.0 (48.3, 71.8)
Gender, female, n (%)	29 (40.3)
APACHE II score, median (Q1, Q3)	22 (17, 29.5)
SOFA score, mean ± SD PSI score on ICU admission	8.4 ± 3.2 110.0 (80.0, 128.0)
Comorbidities, n (%)	
Immunosuppression^*^	27 (37.5)
Cerebral stroke	15 (20.8)
Diabetes	15 (20.8)
Cardiovascular disease	8 (11.1)
Chronic kidney disease	8 (11.1)
COPD	8 (11.1)
Hematopoietic malignancies	7 (9.7)
Postoperative tumor	6 (8.3)
Pulmonary fibrosis/Interstitial lung	5 (6.9)
Autoimmune diseases	4 (5.6)
Bronchiectasis	3 (4.2)
Application of antibiotics before mNGS, n(%)	72 (100)
Application of antifungal agent before mNGS, n(%)	50 (69.4)
Application of antivirals before mNGS, n(%)	24 (33.3)
Hospital stays, days, median (Q1, Q3)	25.0 (15.0, 37.5)
Length of stay in ICU, days, median (Q1, Q3)	20.0 (14.0, 28.0)
Length of stay in ICU before mNGS, median (Q1, Q3)	10.0 (7.0, 14.0)
Duration of mechanical ventilation before mNGS, median (Q1, Q3)	7.0 (7.0, 11.0)
Duration of mechanical ventilation, median (Q1, Q3)	8.0 (4.0, 14.0)
ICU outcome, n (%)	
Improved, n (%)	34 (47.2)
Death, n (%)	38 (52.8)

* Immunosuppression, defined as chemotherapy or neutropenia <1,000 μl during the past 28 days; treatment ≥20 mg corticosteroids daily for ≥14 days; human immunodeficiency virus infection; immunosuppressive therapy after organ or bone marrow transplantation; active tuberculosis. COPD, chronic obstructive pulmonary disease; PSI, Pneumonia Severity Index.

### Comparison of mNGS and CT Pathogen Detection Methods

**Figure 2:**
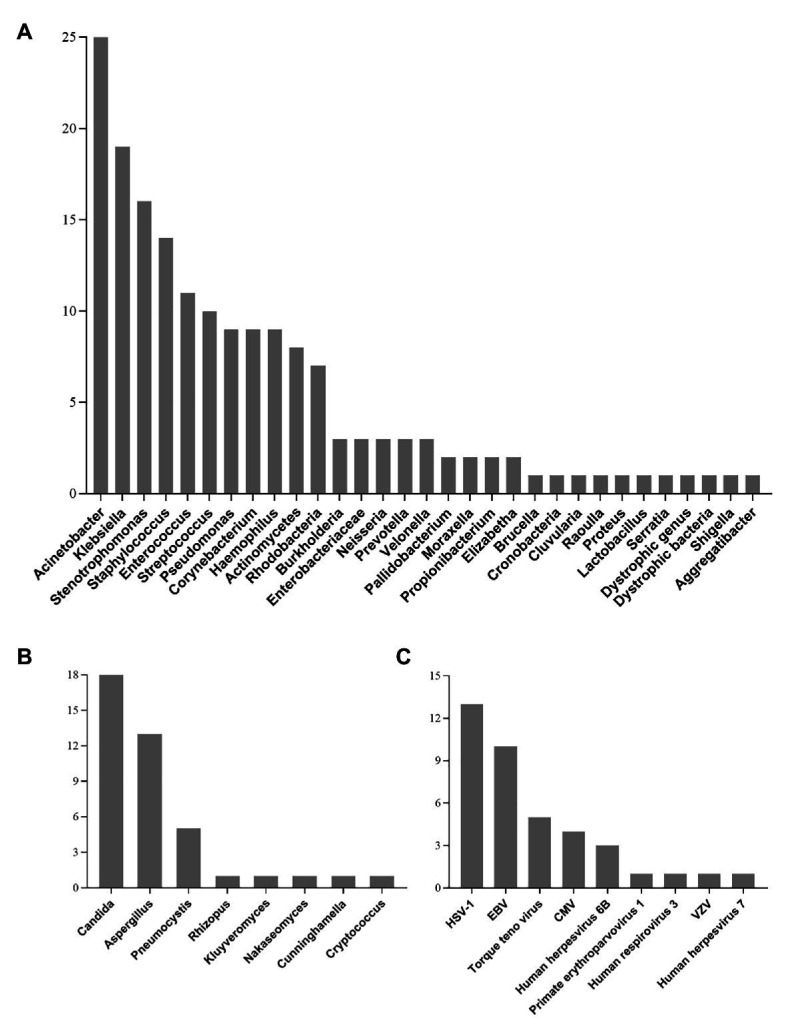
Genus distribution of bacteria **(A)**, fungi **(B),** and virus **(C)** detected by mNGS technique. *Acinetobacter*, *Candida*, and HSV-1 were the most commonly detected bacteria, fungi, and viruses, respectively. HSV-1, Herpes simplex virus 1; EBV, Epstein-Barr virus; CMV, Human cytomegalovirus; VZV, varicella zoster virus.

All detected bacterial and fungal (at genus level) samples, along with viruses are listed in Figure 2. Among the microbes isolated, *Acinetobacter*, *Klebsiella*, and *Stenotrophomonas* were the most frequently detected bacteria. The most commonly detected fungi were *Candida*, *Aspergillus*, and *Pneumocystis*. In addition, a total of 30 patients were virus-positive (41.7%) and the most frequently detected viruses were HSV-1, EBV, and torque teno virus. One case of *Chlamydia* (PT18) and two cases of *Mycoplasma* (PT26 and PT50) were also detected by mNGS (Supplementary Material 3).

**Figure 3:**
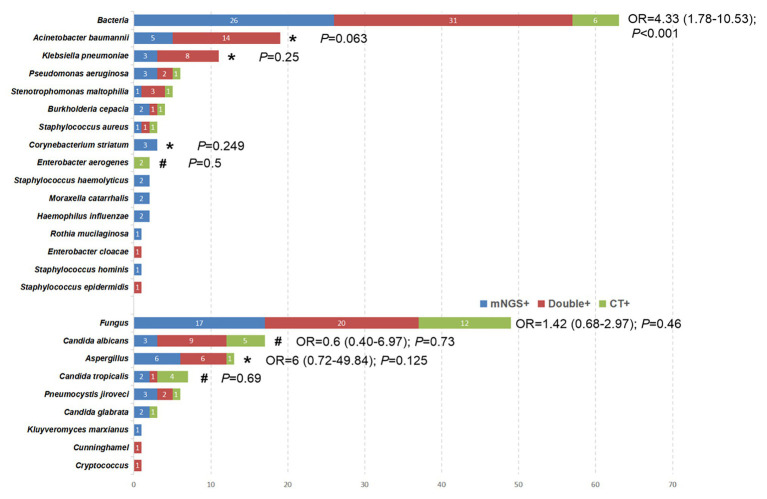
The overlap of positivity between mNGS technique and CT methods for different pathogens. ^*^The pathogens were observed to have a higher positive rate by mNGS than that by CT methods, although the difference was not significant (*p* > 0.05). ^#^The positive rate of CT method was higher than that of mNGS, but the data did not show a significant difference (*p* > 0.05).

*Acinetobacter baumannii* was still the most commonly detected pathogen as determined by the CT method, followed by *Klebsiella pneumoniae* and *Stenotrophomonas maltophilia* (Figure 3). *Candida albicans*, *Aspergillus*, and *Candida tropicalis* were the top three of the most detected fungi as determined by CT methods (Figure 3). Viral detection, including CMV, EBV, HSV-1/2, and parainfluenza virus, was performed by PCR. In the present study, PCR was only performed for viral nucleic acid detection of 19 patients, of which 12 cases yielded positive results. Thus, a comparison between PCR and mNGS for viral detection could not be established. Specifically, the CT method revealed positive detection for six EBV cases, four CMV cases, one case of HSV-1, and one case of parainfluenza virus infections.

The comparison of bacterial and fungal detection by the mNGS and CT methods is shown in Figure 3. The percentage of mNGS-positive samples was significantly higher than that of CT-positive samples with regard to bacterial detection (OR, 4.33; 95% CI, 1.78–10.53; *P* < 0.001), while no significant differences were noted with regard to fungal detection (OR, 1.42; 95% CI, 0.68–2.97; *p* = 0.46). Specifically, *Acinetobacter baumannii* (100% vs. 73.7%; *p* = 0.063), *Klebsiella pneumoniae* (100% vs. 72.7%; *p* = 0.25), *Aspergillus* (92.3% vs. 53.6%; *p* = 0.125), and *Corynebacterium striatum* (100% vs. 0%; *p* = 0.25) exhibited a higher detection rate by the mNGS method compared with that noted by the CT method, although the differences were not significant (*p* > 0.05). However, the percentage of CT-positive samples was higher than that of mNGS in some pathogenic agents [e.g., *Enterobacter aerogenes* (100% vs. 0; *p* = 0.5), *Candida albicans* (82.4% vs. 70.6%; *p* = 0.134), and *Candida tropicalis* (71.4% vs. 42.9%; *p* = 0.68)].

### Diagnostic Performance Comparison of mNGS and CT Methods

Bacterial pathogens were detected by the mNGS method in 55 (76.4%) samples, while the CT-positive samples were 34 (47.2%; Supplementary Material 2). mNGS exhibited a diagnostic sensitivity of 97.1% (95% CI, 93.2–101.0%) and a specificity of 42.1% (95 CI%, 30.7–53.5%) for bacterial detection. The PPV and NPV values of the mNGS method were 60.0% (95% CI, 48.7–71.3%) and 94.1% (95% CI, 88.7–99.6%), respectively, for bacterial detection. With regard to the fungal detection, the results of both methods were positive in 32 cases. mNGS exhibited a diagnostic sensitivity of 71.9% (95% CI, 61.5–82.3%) and a specificity of 77.5% (95% CI, 67.9–87.1%) for fungal detection. The PPV and NPV of the mNGS method for fungal detection were 71.9% (95% CI, 61.5–82.3%) and 77.5% (95% CI, 67.9–87.1%), respectively.

### Identification of Pathogens in CT-Negative Samples by the mNGS Method

For bacterial detection, the mNGS analysis yielded negative or nonspecific results in 16 out of the 38 BALF samples, in which the bacteria were not detected by the CT method (PT1, PT10, PT11, PT14, PT21, PT22, PT25, PT32, PT33, PT46, PT51, PT52, PT57, PT58, PT62, and PT70). In addition, mNGS analysis yielded positive results in 22 CT-negative samples (PT2, PT9, PT12, PT19, PT30, PT36, PT44, PT47, PT50, PT53–56, PT61, PT63, PT64, PT66–69, PT71, and PT72). Among these mNGS-positive samples, only eight samples corresponded to Gram-positive bacteria and the remaining were Gram-negative bacteria. (Supplementary Material 3).

For fungal detection, mNGS analysis yielded positive results in 9 out of 40 BALF samples, which were not found to be positive by the CT method (PT14, PT19, PT22, PT35, PT36, PT60, PT66, PT69, and PT72). The majority of the nine patients underwent immunosuppressive therapy (with the exception of PT19 and PT66). (Supplementary Material 3).

### Identification of Pathogens in CT-Positive Samples by the mNGS Method

For bacteria, the most abundant species detected by the mNGS method was also verified by the CT method in 26 samples. Among PT13, PT23, and PT40, the bacteria identified by the CT methods were not the most abundant species in the mNGS results. Furthermore, we also observed that the CT method identified two types of bacteria in the BALF samples, whereas only one was detected by the mNGS method (PT28 and PT31). Specifically, *Staphylococcus aureus* and *Enterobacter aerogenes* in PT28 and PT31 were not detected by the mNGS method. Conflicting data from the mNGS and CT methods were obtained in the three samples (PT29, PT35, and PT45). In addition, only one patient (PT3) sample yielded mNGS-negative results among CT-positive samples. In this patient, bacterial smear indicated a low number of Gram-negative bacteria, while mNGS results indicated *Actinomycetes* with the unique reads three, which was not interpreted as pathogenic bacteria. (Supplementary Material 3).

For fungi, among the CT-positive samples (32 cases), mNGS analysis yielded negative or non-specific results in nine samples (PT4, PT6, PT15, PT21, PT29, PT33, PT37, PT40, and PT42; Supplementary Material 2). In addition, mNGS analysis produced positive results in 23 out of 32 CT-positive samples. The results of the CT and mNGS methods were consistent in 21 patients (PT1, PT2, PT12, PT18, PT25, PT26, PT31, PT34, PT38, PT41, PT45, PT47, PT50, PT52, PT57–59, PT62, PT63, PT65, and PT68). However, in the PT44 and PT70 samples, conflicting data were obtained from the mNGS and CT results. The CT results of these two patients revealed positive detection for *Candida albicans*, while mNGS identified *Aspergillus* as the most abundant fungus.

### Diagnostic Performance of the Combination of mNGS and CT Methods in Mixed Infection

**Figure 4:**
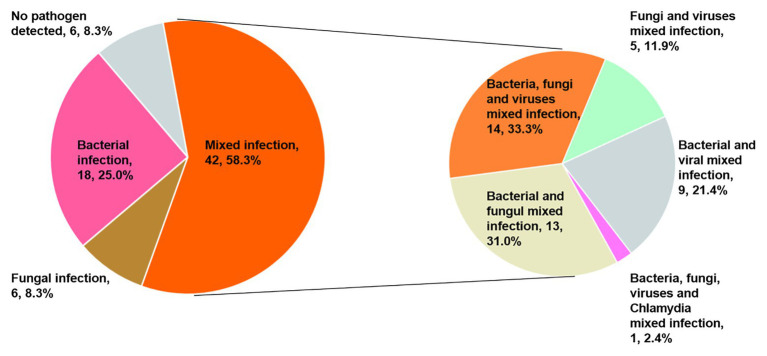
Percentage of patients with mixed infections for various pathogens.

Only 40 patients (55.6%) were diagnosed with a mixed infection using solely the mNGS method. However, when combined with CT detection results, the diagnostic ratio of the mixed infection was increased to 58.3% (42/72). It is interesting to note that 14, 13, 9, 5, and 1 patients were present that could be diagnosed as mixed bacterial-fungal-viral, bacterial-fungal, bacterial-viral, fungal-viral, and bacterial-fungal-viral-*Chlamydia psittaci* infections (Figure 4).

## Discussion

Despite advances in prevention, diagnosis, and treatment of VAP, this disease remains a major contributor to morbidity and mortality of intubated patients. A rapid microbiological diagnosis of VAP facilitates the timely and precise application of antimicrobial therapy. Although the majority of the research and clinical studies have concluded that mNGS exhibits promising results as a novel method for microbial identification, conflicting evidence has also been reported as follows: mNGS may be considered superior to the CT method in microbiological testing. To date, the largest study is that conducted by Qing et al. (Miao et al., 2018), which indicated that the mNGS was not superior to the CT methods for detecting common bacteria, whereas it exhibited a better performance than the CT methods with regard to fungal detection. However, another study reached the opposite conclusion (Xie et al., 2019). This divergence may be attributable to different specimen sources from multiple clinical departments and even different diseases, leading to inconclusive results. Unfortunately, previous studies did not perform subgroup analysis in BALF samples. All the patients enrolled in the present study developed VAP in the ICU and all samples were derived from BALF. The sensitivity, specificity, PPV, and NPV were compared between mNGS and CT methods using a paired comparison and the advantages and disadvantages of the mNGS method were found in the identification of bacterial and fungal detection. Specifically, for bacterial detection, the present study indicated that the percentage of mNGS-positive samples was significantly higher than that of CT-positive samples. Furthermore, *Acinetobacter baumannii*, *Klebsiella pneumoniae*, and *Corynebacterium striatum* exhibited a higher yield rate by the mNGS method compared with that noted by the CT method. Although six bacterial strains were not detected by the mNGS method (Figure 3), the sensitivity and NPV of mNGS with regard to bacterial detection was as high as 97.1 and 94.1%, respectively, which were much higher than those of the CT method. In the PT3 sample, the CT (smear) results indicated a low number of Gram-negative bacteria, which was not detected by the mNGS method. In addition to this sample, two species of bacteria were detected by the CT method, while only one bacterium was detected using the mNGS method in the remaining five samples. Unfortunately, insufficient evidence was present to assess the validity of the detection results. In summary, mNGS could be used as a routine diagnostic tool for bacterial infections, notably for ICU patients, although specific disadvantages were noted, such as false negative detections might be caused by human error. Another finding of the present study was that the mNGS did not outperform the CT method for fungal detection, which was consistent with the conclusion of Yun et al. (Xie et al., 2019). Both detection techniques had their own advantages for the identification of specific fungi. For example, CT exhibited a superior feasibility in detecting *Candida albicans* and *Candida tropicalis*, while mNGS indicated a higher detection rate of *Aspergillus*. Overall, based on specific advantages in the detection of different pathogens, the data suggested that clinicians should take into consideration the CT-results following selection of mNGS.

To date, no uniform standards have been reported regarding the interpretation of CT and mNGS results, it is therefore important to remain cautious when interpreting the mNGS results. The interpretation requires a specific number of unique reads and a relative abundance of the detected pathogens. The higher the number of unique reads and the relative abundance of the detected microorganisms, the higher the possibility of microorganisms to be considered as infectious pathogens (Schlaberg et al., 2017). Following the exclusion of background bacteria, contaminating and colonizing bacteria could be considered as pathogenic pathogens (Schlaberg et al., 2017; Han et al., 2019). We also noticed that the number of unique reads, which were used to identify pathogens, was substantially reduced when contrasting thin-walled pathogens, such as bacteria, with specific thick-walled organisms, such as fungal pathogens. However, the interpretation of mNGS results should not be based solely on reports; most importantly, physicians should analyze these test results in combination with clinical features in order to obtain a comprehensive consideration of bacterial, fungal, or viral detection prior to decision-making on therapeutic choices. We must, however, acknowledge that the clinical misjudgment cannot be completely avoided, even for experienced groups of physicians. On an optimistic note, with the increasing application of mNGS, a large amount of meaningful work is currently being undertaken to help interpret the complex results. Perhaps mNGS in conjunction with histopathologic examinations [rapid on-site evaluation of cytology (ROSE) and endobronchial ultrasound-guided transbronchial needle aspiration (eBUS-TBNA)] may further resolve this issue. It was of particular emphasis in this work that the different diagnostic criteria in the diagnostic guidelines for *Candida* pneumonia in China are comparable to those in foreign countries (Infection group, respiratory branch, Chinese Medical Association, 2007; Japanese Society of Mycology, 2013; Shi, 2018). However, the detection of *Candida* has great clinical significance for VAP patients. Previous study findings suggested that even the colonization state may promote the occurrence of VAP, leading to the emergence of various multidrug-resistant bacterial pathogens and, thus, increasing the length of hospital stays (Azoulay et al., 2006; Nseir et al., 2007; Marie-Soleil et al., 2016). Hence, to provide enough information for clinical decision-making, *Candida* was classified as a “pathogen” in this report, even though these patients cannot be strictly diagnosed as having *Candida* pneumonia (except for PT21; for more information, see Supplementary Material 3).

The majority of the studies that have used the mNGS methods are based on their diagnostic value for specific pathogens (Miao et al., 2018). However, the inconsistent results between the mNGS and CT methods have been rarely reported in the literature. This information may create confusion regarding the choice of treatment. This was also noted in the present study. The possible reasons are as follows: (a) certain BALF samples may have been contaminated during the collection process; (b) the operation process of mNGS exhibited high demands on the operating environment and the samples were more likely to be contaminated by nucleic acids in the laboratory; however, in general, the number of unique reads of contaminating bacteria was low; (c) specific pathogens were considerably difficult to culture, such as the *Corynebacterium striatum* (PT35, PT68, and PT72), due to misidentification caused by CT methods that mainly detect normal colonization of human skin and mucous membranes (Díez-Aguilar et al., 2013; Dickson et al., 2014); however, mNGS may be more reflective of the real infection. Therefore, when faced with inconsistent test results, it is suggested to repeat CT or mNGS detection methods in order to perform reliable judgments based on microbial characteristics and clinical data. For example, the PT44 fungal culture indicated *Candida albicans*, while mNGS identified *Aspergillus* as the most abundant fungal pathogen. Following multiple sputum smear/culture examinations, *Aspergillus* infection was subsequently confirmed by culture results. The final diagnosis of allergic bronchopulmonary aspergillosis was established based on the comprehensive assessment of multiple test results and clinical manifestations.

In the present study, 30 cases of viruses were also detected by mNGS and the majority of them were co-infected with bacteria and fungi. In recent years, clinicians have become more aware of the effects of viral co-infection on the development of pneumonia. Voiriot et al. used PCR analysis and demonstrated that patients with viral-bacterial co-infection conferred a significantly worse prognosis (Voiriot et al., 2016). However, physicians may not be fully aware of this problem in their daily clinical work. This is because the detection of viral nucleic acids by PCR is not performed on every patient. Furthermore, the limited detection range of the PCR kits may not be accurate in identifying the viral strains present in the samples. Notably, the mNGS method exhibited apparent advantages in the simultaneous detection of bacteria, fungi, and viruses compared with the CT method (Wang et al., 2019). By analyzing the results of the mNGS and CT methods, the proportion of mixed infections in VAP was higher than originally thought. In addition to the mixed viral infections noted in 29 patients [only in one patient (PT21), the EB virus was identified to be in a latent state by the clinician], 13 patients were diagnosed as a mixed bacterial and fungal infection. Although the association of the detected virus with the observed clinical symptoms is considerably difficult to establish, notably for viruses not commonly detected in humans, the mNGS method may aid the clinicians to identify pulmonary mixed infections in ICU patients and make rational treatment decisions.

In the present study, the turnaround time of sequencing was 24–48 h. As a result of the localization of the hospital site sequencing platform, this time has been reduced to <24 h. In contrast, the average feedback time for pathogen culture is 2–5 days for bacteria or fungi and 45 days for TB. Although the current sequencing cost ($500 per sample) is higher than any other conventional testing method (hospital charges are $60 for bacterial culture, $10 for BALF smear, $20 for Grocott’s methenamine staining, $150 for TB-spot, and $20 for virus PCR test), as seen in the present work, the positive rate for conventional testing is still relatively low and repeated tests are usually needed. Before mNGS was available, culture and smear tests were performed 5.4 ± 2.1 times; thus, the total cost of pathogen diagnosis was equivalent to that of a single mNGS. Therefore, we concluded that the conventional testing methods were not much cheaper than mNGS, especially when they were used simultaneously for pathogen screening.

The present study contains certain limitations. The patients enrolled had only undergone DNA sequencing. RNA sequencing was not performed simultaneously, which may lead to false negative detection of certain pathogens. Due to the retrospective nature of the study, viruses were occasionally detected in certain BALF samples using the PCR method. Moreover, the viruses detected by the mNGS method were not diagnostically confirmed. It is important to note that the interpretation of the mNGS results depends on the subjective judgment of the clinician. In addition, significant differences were present in the sequencing processes and in the quality of the reporting forms, which may lead to a bias in the mNGS results.

Overall, in patients who developed VAP, the application of the mNGS method for BALF testing may improve the sensitivity and specificity of pathogen detection, notably for bacteria. In addition, mNGS is a promising technique for the detection of mixed infections. However, the interpretation of mNGS data should be combined with CT results and clinical data due to the lack of unified diagnostic criteria.

## Data Availability Statement

The original contributions presented in the study are included in the article/Supplementary Material, further inquiries can be directed to the corresponding authors.

## Ethics Statement

Ethical review and approval was not required for the study on human participants in accordance with the local legislation and institutional requirements. Written informed consent to participate in this study was provided by the participants’ legal guardian/next of kin. Written informed consent was not obtained from the individual(s) for the publication of any potentially identifiable images or data included in this article.

## Author Contributions

AP contributes substance to ideas and design. XwF, QM, and XqF draft the paper. CZ and TY conduct statistical analysis on the data. LZ and SG give a lot of assistance and revises manuscript. All authors contributed to the article and approved the submitted version.

### Conflict of Interest

The authors declare that the research was conducted in the absence of any commercial or financial relationships that could be construed as a potential conflict of interest.

## Footnotes

^1^https://ftp.ncbi.nlm.nih.gov/genomes/

## Supplementary Material

Data Sheet 1 Original data.

Data Sheet 2 Diagnostic performance comparison of mNGS and CT methods.

Data Sheet 3 Supplementary results.
