# Supplementary material for: Diagnostic Value of Metagenomic Next-Generation Sequencing for the Detection of Pathogens in Bronchoalveolar Lavage Fluid in Ventilator-Associated Pneumonia Patients
Source: Front Microbiol. 2020 Dec 1;11:599756. doi: 10.3389/fmicb.2020.599756 (PMC7736608; doi:10.3389/fmicb.2020.599756)
Supplement: Data Sheet 2 [file Data_Sheet_2.docx]

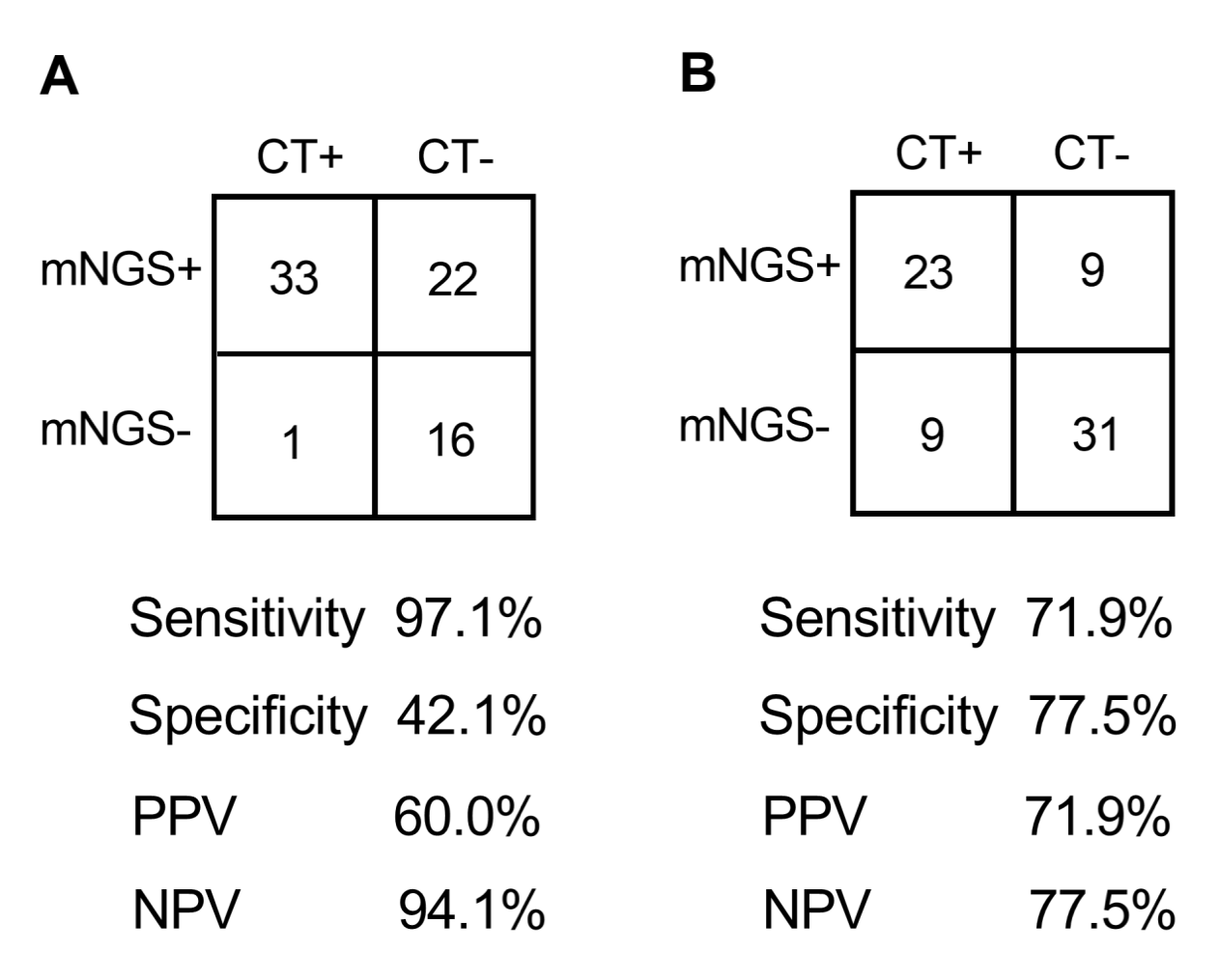


Contingency tables formatted in a 2X2 manner showing the respective diagnostic performance of mNGS and CT detecting for bacteria (A) and fungi (B). CT, conventional testing; PPV, positive predictive value; NPV, negative predictive value.
