# Supplementary material for: Diagnostic Value of Metagenomic Next-Generation Sequencing for the Detection of Pathogens in Bronchoalveolar Lavage Fluid in Ventilator-Associated Pneumonia Patients
Source: Front Microbiol. 2020 Dec 1;11:599756. doi: 10.3389/fmicb.2020.599756 (PMC7736608; doi:10.3389/fmicb.2020.599756)
Supplement: Data Sheet 3 [file Data_Sheet_3.docx]

**Supplementary results**

**More details of PT18, PT26 and PT50**

**PT18:** in PT18, *Chlamydia psittaci* (relative abundance 77.38%) was detected by mNGS and the unique reads were 559. Final diagnosis was bacterial-fungal-viral-Chlamydia psittaci co-infections.

**PT26 and PT50:** *Oral mycoplasma* was detected using mNGS in PT26 and PT50 with unique reads of 23 and 3, respectively. However, the detection of *oral mycoplasma* was not interpreted as a pathogen since it is a microbe that belongs to the normal oral pathogen family.

**Identification of pathogens in CT-negative samples by the mNGS method**

**Negative or nonspecific results of mNGS (Bacteria).** The mNGS analysis yielded negative or nonspecific results in 16 out of the 38 BALF samples, in which the bacteria were not detected by the CT method (PT1, PT10, PT11, PT14, PT21, PT22, PT25, PT32, PT33, PT46, PT51, PT52, PT57, PT58, PT62, and PT70). A total of 7 samples were negative as determined by both methods and the bacterial nucleic acid was detected by the mNGS method in the remaining 9 samples. However, these bacteria were detected with low unique reads (range from 3 to 649) and relative abundance (ranging from 0.16% to 18.86%). Moreover, they were nonspecific for VAP and then they were not interpreted as infectious pathogens.

**Positive results of mNGS (Bacteria).** mNGS analysis yielded positive results in 22 CT-negative samples (PT2, PT9, PT12, PT19, PT30, PT36, PT44, PT47, PT50, PT53-56, PT61, PT63, PT64, PT66-69, PT71, PT72). Among these mNGS-positive samples, only 8 samples corresponded to gram-positive bacteria (*Rothia* [PT2], *Staphylococcus* [PT64, PT66, PT67, PT69], *Corynebacterium* [PT68, PT72], *Streptococcus* [PT71]) and the remaining were gram-negative bacteria. The most commonly detected gram-negative bacteria were *Acinetobacter* (PT12, PT19, PT36, PT47, PT63), followed by *Haemophilus* (PT9, PT56), *Klebsiella* (PT30, PT55), *Pseudomonas* (PT44, PT61), *Burkholderia* (PT50) and *Moraxella* (PT53, PT54).

**Positive results of mNGS (Fungi).** mNGS analysis yielded positive results in 9 out of 40 BALF samples, which were not found to be positive by the CT method. The majority of the 9 patients underwent immunosuppressive therapy (with the exception of PT19 and PT66). The most commonly detected fungi were *Candida* (PT19, PT35, PT36, PT60, PT66, and PT69), followed by *Aspergillus* (PT19, PT60, and PT62) and *Pneumocystis* (PT14, PT22).

**Identification of pathogens in CT-positive samples by the mNGS method**

**For bacteria, the most abundant species detected by the mNGS method was also verified by the CT method in 26 samples.** Two types of bacteria were detected in PT16 and PT34 by the culture method. In PT16, bacterial culture indicated *Klebsiella pneumoniae* and *Acinetobacter baumannii*, which ranked first and third in the bacterial species. The bacterial species that ranked second was *Corynebacterium striatum*, which was difficult to test in a routine microbiology laboratory. In PT34, *Acinetobacter baumannii* and *Enterobacter cloacae* ranked first and second and were also concomitantly detected by the CT method.

**The bacteria identified by the CT methods were not the most abundant species in the mNGS results.** In PT13, CT indicated *Acinetobacter baumannii* and *Streptomonas maltophilia*, which ranked second and third in the mNGS results and demonstrated higher than 50 unique reads. However, *Burkholderia,* which ranked No. 1, was not detected by the CT method. In PT23, *Acinetobacter baumannii* was detected by the CT method (only 21 reads at the genus level, ranking second), while mNGS data indicated that *Corynebacterium striatum* was the most abundant species with 37.41% relative abundance. *Stenotrophomonas maltophilia* was identified by the CT method in PT40. mNGS analysis at the species level detected *Stenotrophomonas maltophilia* with 27.5% relative abundance and 130 unique reads, which conformed to the threshold. However, the top two bacterial strains (Palebacterium and Acinetobacter) were not detected by the CT method.

**The CT method identified 2 types of bacteria in the BALF samples, whereas only 1 was detected by the mNGS method (PT28, PT31).** Specifically, *Staphylococcus aureus* and *Enterobacter aerogenes* in PT28 and PT31 were not detected by the mNGS method. (Supplementary table 1)

**Conflicting data from the mNGS and CT methods (PT29, PT35, and PT45)**. In PT29, *Enterobacter aerogenes* was detected in BALF and blood samples several times. However, mNGS results indicated detection of *Klebsiella pneumoniae*. Additional analysis led to the conclusion that *Enterobacter aerogenes* was not detected due to human error. PT35 corresponded to a 29-year-old female subject who had been admitted to the hospital due to bronchiectasis and lung infection following double lung transplantation. *Burkholderiacepacia* is a bacterial species, which was detected in the bacterial culture and was not specifically detected by the mNGS method. The mNGS method identified *Pseudomonas aeruginosa* as the most abundant species in bacteria. Furthermore, *Pseudomonas aeruginosa* was repeatedly detected in PT45 by the culture method, whereas the only positive bacteria identified in that sample by the mNGS method was *Corynebacterium striata*.

**Candida pneumonia**

PT21 was diagnosed as ANCA-associated vasculitis and received combination immunosuppressive therapy, including glucocorticoids and cyclophosphamide. Subsequently, *Candida* was detected in pleural effusion for many times, in this case the diagnostic criteria of Candida pneumonia was fulfilled.
